# Supplementary material for: Parametric evaluation of impedance curve in radiofrequency ablation: A quantitative description of the asymmetry and dynamic variation of impedance in bovine ex vivo model
Source: PLoS One. 2021 Jan 15;16(1):e0245145. doi: 10.1371/journal.pone.0245145 (PMC7810295; doi:10.1371/journal.pone.0245145)
Supplement: Attachment — Submitted filename: Response to Reviewers.pdf [file pone.0245145.s002.pdf]

Response to Additional Editor Comments:

(**E**ditor) While characterization of electrical properties of bovine liver is an important task there are several critical issues and concerns the authors should address before initiating the review process. Below are my major concerns:

1. The authors should use the term electrical resistance and impedance because there are several meanings of resistance.

(**A**nswer): We are grateful for the suggestion of the possible application of both terms, considering that only when using the real part in the analysis the term resistance can be used, that is, it is treated as purely resistive. However, according to the more detailed explanation below, the group opted to standardize the use of the term impedance in the manuscript.

2. The authors have a mix of the terms “resistance” and “impedance.” It is common to use resistance for DC and impedance for AC current. The word “resistance” is in the title but the y-axis in Fig. 2 is labeled “Impedance.”

A: We welcomed the suggestion to standardize the term impedance in the study applied to RFA. Impedance is composed of a real and imaginary part, the latter being the reactive component in the system. From the radiofrequency point of view, there are two elements: a purely resistive element associated with a capacitive reactance. This indicates that there is a phase shift between the voltage and current applied during the RFA. When using only the real part in the analysis, the term resistance can be used, that is, it is treated as purely resistive. However, in this case it is consistent to use impedance in agreement with the application of the RFA technique with alternating current, even though only the purely resistive part, sufficient to parameterize satisfactory performance indexes for clinical application, is analyzed. Thus, we have readjusted the title of the manuscript so that it is consistent with what has already been established in literature.

3. The electrical impedance of ex vivo human tissues were comprehensively studied in previous literature: See: Jossinet J (1996). Variability of impedivity in normal and pathological breast tissue. Med.& Biol. Eng. & Comput. 34:346—350 and Jossinet J (1998). The impedivity of freshly excised human breast tissue. Physiological Measurements 19:61—75. The authors miss these important references.

A: We are grateful for the suggestion to include the reference of the study carried out on normal and tumor human tissues, in addition to others, allowing an overview of the basic parameters for impedance assessment. With these data, there was an enrichment of the text about the basic concepts on impedance in human tissues present in literature, in addition to those existing in liver tissues already brought in the text and more current. The aforementioned author carried out a comprehensive assessment regarding the behavior of the variable in a frequency range and with diversity of tissues for comparison.

4. Fig. 2 shows only one curve how impedance depends on time. I suggest showing all curves in five plots corresponding to 5 groups. How easy is to detect the points on the curve displayed in Fig. 2? My impression is that in some curves the identification of those points is not straightforward and therefore this method is prone to large variation and imprecise (see the next item).

A: We appreciate the suggestion to include the graph of the impedance profiles. They actually made it clear what the profile is in each group. As suggested, the group added a subsection "*Obtained impedance curves*" in the results section and another graph (Fig. 4) showing the impedance profile divided by group. In fact, there is a great variability of the curves that is expected in RFA, this dispersion being characteristic of the RFA process. Several factors contribute to this, such as the temperature of the tissue being treated, which is affected by the temperature rise caused by the ablation, the volume of tissue submitted to the ablative process, and the dissipative effect caused by the presence of blood vessels, among other factors. Thus, it is a challenge to obtain a model that incorporates all these dynamic variables. The proposed analysis aims to allow the clinician a preliminary view based on simple parameters to be calculated.

Contrary to the first impression obtained that the points are not easy to identify, the curves shown allow the visualization and determination of the points for calculating the indices in each evaluated group.

5. In general, I'm not impressed with the suggested method of characterizing the impedance of bovine liver due to high scatter: most results are not statistically significant. I suggest the authors to investigate two approaches to improve the comparison across groups: (a) two-way ANOVA when all points on the curve are used simultaneously for group discrimination, (b) nonlinear modeling approach as outlined in ref [20].

A: Thank you very much for the comment. The dispersion of the data is something that cannot be eliminated as explained in the previous point, being a characteristic of RFA. The group took great care in minimizing dispersions and biases in the data, either in the experimental execution or in the treatment of the data during the analyses, evaluating the influences of outliers in the general result.

The null hypothesis of this work is that the proposed parameters and indices are not influenced when subjected to different types of solutions and temperatures, that is, the means of the effects are the same in each group. Thus, it is expected that in the results the p-values are not significant. The exception is for the correlation between indices and volumes.

a) As suggested, we performed a two-way ANOVA with the factors being the type of solution (SALINE, DEIONIZED and the RFA control) and the temperature of the solution (ROOM TEMPERATURE, REFRIGERATED and the RFA control). The results corroborate with the analyses carried out in the previous manuscript and demonstrate that the indices, despite the dispersion of the data, present similar values in the analyzed factors. These findings were incorporated into the manuscript along with 3 new images (Figs 11, 12 and 13) and Table 1 was revised, which now points to the mean estimates and their confidence intervals instead of the previous minimum and maximum values, in addition to a new one table for the analyzed factors (Table 2).
b) The suggestion to use a non-linear approach is a great one and has already been carried out by the group in a previously published work. Unlike the nonlinear approach that seeks to describe the impedance dynamics as a function of the procedure time, this work seeks to standardize notable points

on the impedance curve. We believe that the present work is different because it is easy to estimate without using computational resources.
