# Supplementary material for: Parametric evaluation of impedance curve in radiofrequency ablation: A quantitative description of the asymmetry and dynamic variation of impedance in bovine ex vivo model
Source: PLoS One. 2021 Jan 15;16(1):e0245145. doi: 10.1371/journal.pone.0245145 (PMC7810295; doi:10.1371/journal.pone.0245145)
Supplement: Attachment — Submitted filename: Response to Reviewers.pdf [file pone.0245145.s003.pdf]

Dear Reviewer(s),

It is a great satisfaction for our group to forward this work after careful analysis and corrections.

We appreciate the relevant comments made to improve the work done. We inform you that all suggestions for changes and additions have been accepted and incorporated into the text.

We are available to address any other issues that may arise.

Regards,

Reviewer #1: General Comment:

This paper presents an attractive impedance-based method for evaluating the radiofrequency ablation in biological tissue. The authors address the problem by parameterizing the impedance measurements using three performance indices relating the sample conditions. The paper details the experimental conditions and presents useful results based on experimental data. The paper has an adequate structure is mostly clear. I feel that the paper has value to be published by fixing some quantitative and qualitative details. Some suggestions are proposed to improve the paper readability and quality.

Comment 1:

Line 43. The authors claim the impedance of the tissues and a circuit. There are some observations here.

1) Clarify that you are talking about electrical impedance.

A: Thank you for the suggestion. We have completely modified the passage mentioned. We have added a description of the tissue impedance and provided references.

2) Formally, the impedance is not an intrinsic electrical property of the tissue as the conductivity and permittivity. In such a sense, the impedance is a generalized property that derives from effective conductivity and permittivity from multiple layers (fat, blood, water. etc). It will be useful to clarify this situation by adding a reference (eg. https://doi.org/10.1016/j.electacta.2018.04.167).

A: We greatly appreciate your relevant comment. We have fully accepted your suggestion and rewrote the entire excerpt including the valuable reference provided.

3) What circuit does the authors are referring? Is it an equivalent circuit model? Again, this latter is a macroscopic approximation of the effective conductivity and permittivity. You could use the same reference of the comment above.

A: We appreciate your comment and suggestion. We clarify that the mentioned circuit corresponds to the electrical system formed by the RF generator, active electrode,

the target tissue, dermis, epidermis, muscles, and the return electrode, as shown in Figure 1 [5]. Thus, the impedance that is obtained by the RFA device is the equivalent impedance of the elements of this electrical circuit. As this is an *ex vivo* study, the influences of the dermis, epidermis and muscles were disregarded.

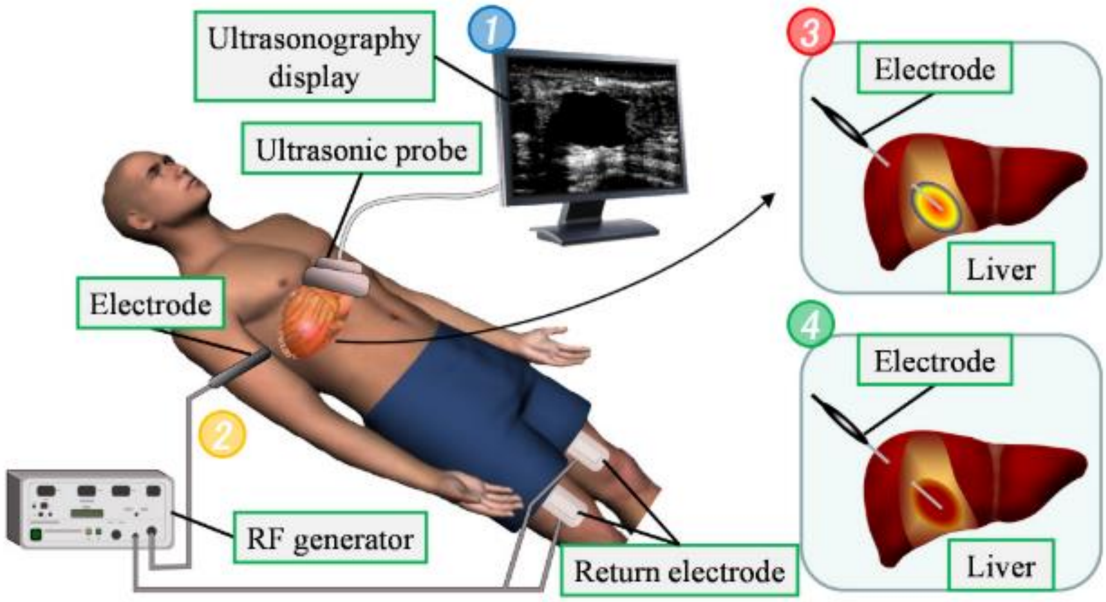

Fonte: RFA procedure [5].

Comment 2:

Line 62. Typo: "though" instead of "through".

A: We appreciate the comment. We sent the manuscript for professional spell checking and the term was corrected. We also carefully reviewed the manuscript for other typographical errors.

Comment 3:

Line 86. Use italic font for "ex vivo".

A: We appreciate the comment. The term is now presented in italics as suggested.

Comment 4:

In Figure 1. The label for the temperature sensor is incorrect, should be "thermocouple" instead of "thermopar".

A: We appreciate the comment. That was a translation error. The correct term was added to the image.

Comment 5:

In Methods section. The authors talk about the experimental setup and equipment. It is not clear, at least for me, how the impedance measurement was done? By inspection of Figure 1, there is solely one electrode coming from the RFA. The authors should explain briefly, in the manuscript, which is the transducing mechanism for the impedance readings, owing that is a key concept of the paper.

A**:** Your comment was very helpful and we are grateful that you have noticed this error in the description of the impedance measurement. In fact, this is a key point that was not described in the manuscript. Now, we give details of this measurement made internally by the equipment using the active electrode and the return electrode. The equipment provides the parameters: applied voltage, current, impedance and duration of the procedure in a .csv file (comma-separated values). The parameters and respective performance indexes were calculated based on this file. We also remade Figure 1 of the experimental setup to better illustrate the procedure.

Comment 6:

In the abstract, the word “fall” is missing for the parameter (ROF).

A: We appreciate the comment and inform you that entire manuscript has been sent back to a specialized translation service.

Comment 7:

In Equations (2), (3) and (4) the subscript “minimum” has a typo, it says "mínimum" (accent). Please, thoroughly revise the whole manuscript for typos.

A: We appreciate the comment and inform you that the authors have reviewed the manuscript thoroughly in search of errors of this nature.

Comment 8:

The caption of Figure 3 talks about x,y,z axes, however in the Figure itself they don´t appear. Add a reference coordinate system in the Figure to improve the readability.

A: We appreciate your suggestion! We added a coordinate system to the image as suggested.

Comment 9:

Due to the work presents a lot of statistical analysis from experimental data. It will be quite useful for the readership to extent the Statistical Analysis section. Instead of only set forth which software and methods are used, the authors should explain in detail why and how the statistical methods were selected.

A: We appreciate your comment about the lack of clarity in the statistical analysis section and agree with your comments.

In order to clarify the way in which statistical analysis was conducted, we rewrote the section on statistical methods giving more details of the computational tools used and statistical analysis.

We specified which STATS v.3.6.0 and LAWSTAT v3.2 packages of R were used.

We also clarified that two ANOVAS were conducted (one-way and two-way). One-way ANOVA was conducted to assess the influence on the defined control and experimental groups (PUERRF, SALINE23, SALINE5, DEIO23 and DEIO5) considering only one factor and one level. Subsequently, two-way ANOVA was used to assess 2 factors with two levels in each and two levels in each factor (deionized water or saline solution and room or refrigerated temperature). Thus, ANOVA only indicates that there is a difference in the groups under analysis, not indicating which group is different. Thus, the *post-hoc* Tukey HSD test was used to determine the divergent group.

We emphasize that the bootstrapping resampling method aims at ensuring that the hypotheses of application of ANOVA (normality and homoscedasticity) are met, and we have detailed how the bootstrapping method was defined and used by adding references to the text.

Comment 10:

In the caption of Figure 5, the variable is missing after the phrase “Performance index”. Moreover, the last sentence is difficult to read.

A: We appreciate the comment. The mathematical symbol used δ (delta) was corrupted by the submission platform. We checked the last section and there was an error in the translation. We have corrected the text.

Comment 11.

The sentence in line 194 has grammatical errors. Check spelling, grammar and typos along the whole manuscript.

A: We appreciate the comment and inform you that the entire manuscript has been sent back to a specialized translation service.

Comment 12.

In the Discussion, the ideas therein are certainly true; however, it shows some mixed concepts and unclear ideas. For instance,

1) Up to this point of the paper, the authors had not introduced neither linear nor non-lineal modeling of the electrical resistivity. It thus could confuse the readership owing that the work is supposed to be based on “linear” modeling by statistics.

It will be quite useful, again, to mention why they used linear statistical models instead of non-linear.

A: Using a non-linear approach is a great approach which was confirmed by the group in a previously published work. Unlike the non-linear approach, which allows to describe the impedance dynamics as a function of the procedure time and other dynamic parameters, here we seek to standardize notable points on the impedance curve. We believe that the present study is different because it allows an easy estimation without the need to use computational resources.

We agree with your suggestion to mention that it is a linear statistical model and highlight the contribution of this approach to the detriment of the non-linear approach and have included it at the end of the introduction.

References

1. Burton C. Heavy tailed distributions of effect sizes in systematic reviews of complex interventions. PLoS One. 2012;7. doi:10.1371/journal.pone.0034222
2. Bjerkli IH, Laurvik H, Nginamau ES, Søland TM, Costea D, Hov H, et al. Tumor budding score predicts lymph node status in oral tongue squamous cell carcinoma and should be included in the pathology report. PLoS One. 2020;15: 1–13. doi:10.1371/journal.pone.0239783
3. Wallisch C, Dunkler D, Rauch G, de Bin R, Heinze G. Selection of variables for multivariable models: Opportunities and limitations in quantifying model stability by resampling. Stat Med. 2020; 1–13. doi:10.1002/sim.8779
4. Zaryab M, Singh-Moon RP, Hendon CP. Robust classification of contact orientation between tissue and an integrated spectroscopy and radiofrequency ablation catheter. Diagnostic Ther Appl Light Cardiol. 2017;10042: 100420O. doi:10.1117/12.2251654
5. Isobe Y, Watanabe H, Yamazaki N, Lu X, Kobayashi Y, Miyashita T, et al. Real-time temperature control system based on the finite element method for liver radiofrequency ablation: Effect of the time interval on control. Conf Proc IEEE Eng Med Biol Soc. 2013;2013: 392–6. doi:10.1109/EMBC.2013.6609519
